# Perioperative Risk Assessment of Patients Using the MyRISK Digital Score Completed Before the Preanesthetic Consultation: Prospective Observational Study

**DOI:** 10.2196/39044

**Published:** 2023-01-16

**Authors:** Fabrice Ferré, Rodolphe Laurent, Philippine Furelau, Emmanuel Doumard, Anne Ferrier, Laetitia Bosch, Cyndie Ba, Rémi Menut, Matt Kurrek, Thomas Geeraerts, Antoine Piau, Vincent Minville

**Affiliations:** 1 Département d’Anesthésie-Réanimation Hôpital Pierre-Paul Riquet Centre Hospitalier Universitaire Purpan Toulouse France; 2 Institut de Recherche en Informatique de Toulouse Université Toulouse III Paul Sabatier Toulouse France; 3 Department of Anesthesia University of Toronto Toronto, ON Canada; 4 Département de Gériatrie Centre Hospitalier Universitaire Rangueil Toulouse France

**Keywords:** chatbot, digital health, preanesthetic consultation, perioperative risk, machine learning, mobile phone

## Abstract

**Background:**

The ongoing COVID-19 pandemic has highlighted the potential of digital health solutions to adapt the organization of care in a crisis context.

**Objective:**

Our aim was to describe the relationship between the *MyRISK* score, derived from self-reported data collected by a chatbot before the preanesthetic consultation, and the occurrence of postoperative complications.

**Methods:**

This was a single-center prospective observational study that included 401 patients. The 16 items composing the MyRISK score were selected using the Delphi method. An algorithm was used to stratify patients with low (*green*), intermediate (*orange*), and high (*red*) risk. The primary end point concerned postoperative complications occurring in the first 6 months after surgery (composite criterion), collected by telephone and by consulting the electronic medical database. A logistic regression analysis was carried out to identify the explanatory variables associated with the complications. A machine learning model was trained to predict the MyRISK score using a larger data set of 1823 patients classified as *green* or *red* to reclassify individuals classified as *orange* as either *modified green* or *modified red*. User satisfaction and usability were assessed.

**Results:**

Of the 389 patients analyzed for the primary end point, 16 (4.1%) experienced a postoperative complication. A *red* score was independently associated with postoperative complications (odds ratio 5.9, 95% CI 1.5-22.3; *P*=.009). A *modified red* score was strongly correlated with postoperative complications (odds ratio 21.8, 95% CI 2.8-171.5; *P*=.003) and predicted postoperative complications with high sensitivity (94%) and high negative predictive value (99%) but with low specificity (49%) and very low positive predictive value (7%; area under the receiver operating characteristic curve=0.71). Patient satisfaction numeric rating scale and system usability scale median scores were 8.0 (IQR 7.0-9.0) out of 10 and 90.0 (IQR 82.5-95.0) out of 100, respectively.

**Conclusions:**

The MyRISK digital perioperative risk score established before the preanesthetic consultation was independently associated with the occurrence of postoperative complications. Its negative predictive strength was increased using a machine learning model to reclassify patients identified as being at intermediate risk. This reliable numerical categorization could be used to objectively refer patients with low risk to teleconsultation.

## Introduction

### Background

In France, the process of a patient undergoing elective surgery includes several essential steps such as the surgical consultation, preanesthetic consultation (PAC), and preanesthetic visit [1]. The decree of December 5, 1994, explicitly states that an anesthesiologist should carry out the PAC [2]. This consultation contributes to the preanesthetic evaluation of the patient’s health status, justifying the prescription of complementary examinations (eg, laboratory tests) and any specialized consultations that allow a perioperative risk assessment formalized by the American Society of Anesthesiologists (ASA) score [3]. This perioperative risk evaluation is used, for example, to determine a patient’s eligibility for an ambulatory care pathway, an enhanced recovery after surgery program or, conversely, a postoperative stay in the intensive care unit [4,5]. As it stands, this state-of-the-art evaluation requires medical expertise.

Beyond the low reproducibility of the ASA score [6,7], the perioperative risk global assessment during the PAC is not well standardized and may be incomplete, especially when consultation time is limited. This is why in some anesthesia teams, patients are asked to complete a questionnaire in paper form before their PAC, allowing them to specify, for example, their past medical and surgical history or their usual treatments. The patient is then asked to hand the completed questionnaire to the anesthetist during the PAC [8]. However, electronic patient-reported outcome measures offer many advantages over paper-based collection [9-13]: preferred modality; (directly) visualized results; higher data quality and response rate; decreased completion time; facilitates patient-clinician communication, improving the decision-making process; and so on. With regard to the preanesthetic questionnaire, the digital version is considered more efficient than the paper form [14]. Moreover, it has been shown that the quality of perioperative care can be improved by a digitalized preoperative information and assessment program [14-16], particularly through automatic reminders or clinical decision support. Health digital tools are therefore definitely of major interest in the perioperative setting.

### Objectives

In 2020, the COVID-19–related restrictions accelerated the implementation of organizational digital health innovations. In the context of the COVID-19 crisis, the anesthesia department at the Toulouse University Hospital in Purpan, Toulouse, France, decided to digitalize the PAC by implementing teleconsultations (*as much as possible* to reduce interpersonal contact) and a digital conversational agent (aka chatbot) that allowed collection of medical data before the PAC. An approach assessing the relevance of the data collected as well as user satisfaction seemed essential to validate the sustainable use of this digital tool. The main objective of this study was to demonstrate that our chatbot was able to stratify patients according to their perioperative risk level. We hypothesized that the *MyRISK* perioperative risk score, established before the PAC according to a predefined algorithm based on data collected digitally, was correlated with the occurrence of postoperative complications at 6 months. Our secondary objectives were to improve the prognostic predictive value of this score using a machine learning model to reclassify patients classified as intermediate risk and assess patients’ and physicians’ satisfaction when using this digital health tool.

## Methods

### Experimental Design

This single-center prospective observational study was conducted in the anesthesia department of the Toulouse University Hospital. To our knowledge, the correlation with postoperative complications of a digital score based on self-reported medical data has never been described. Thus, no assumptions could be made regarding the relative risk and the positive and negative predictive values of being classified as high perioperative risk by the MyRISK score. Given the relatively low postoperative complication rate after scheduled orthopedic surgery (almost 5% [17]), we estimated that approximately 500 patients should be included to meet our objectives (based on expert opinion).

### Population

All patients aged >18 years who were scheduled for orthopedic surgery at the Toulouse University Hospital between June 1, 2020, and October 31, 2020, were eligible. The exclusion criteria were the presence of a protection regime for adults (guardianship, curatorship, or safeguard of justice), patients who did not speak French, the presence of a major sensory handicap (blindness or deafness) compromising the comprehension of the information, patients who did not complete the digital questionnaire through the chatbot (this criterion was considered a refusal to participate), and patients who expressed their opposition to participating in this study.

### MyRISK Score

**Figure 1:**
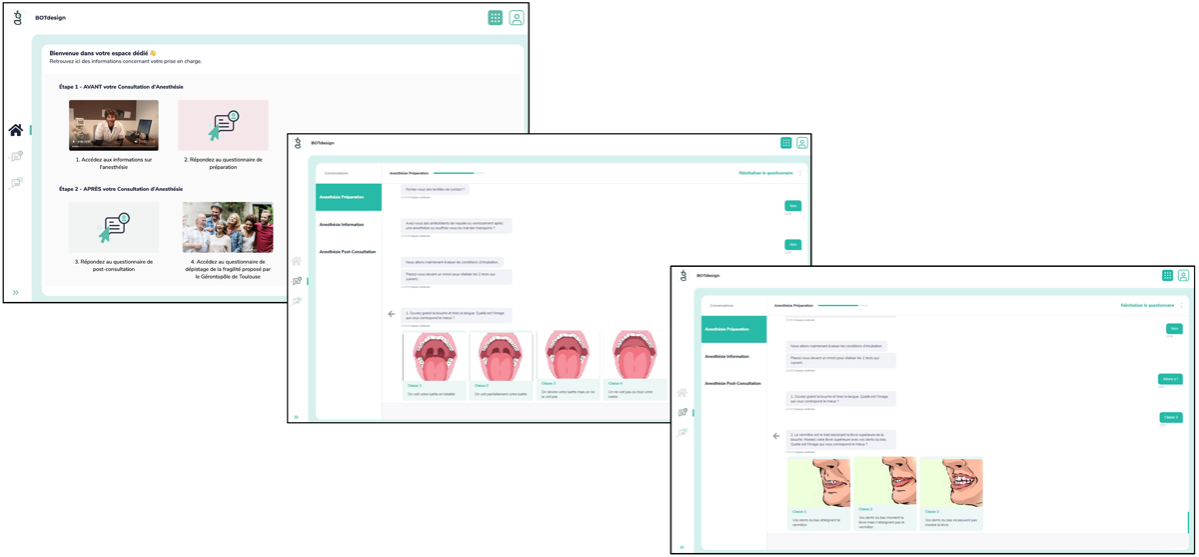
Screenshots of the digital conversational agent Medical Assistant Experience (BOTdesign, Toulouse, France). The patient is asked to complete a self-assessment of its predictive criteria for difficult intubation (Mallampati and upper lip bite tests).

The preanesthetic digital conversational agent, *Medical Assistant Experience* (*MAX*), was developed by 2 anesthetists of the Toulouse University Hospital (FF and VM) in collaboration with a company that creates secure health companions (BOTdesign, Toulouse, France; Figure 1; Multimedia Appendices 1 and 2). Its content was based on the preexisting paper form questionnaire with the addition of anesthetic items considered relevant, such as those allowing the calculation of perioperative scores published in the literature. As an example, we can cite the calculation of the Amsterdam Preoperative Anxiety and Information Scale score [18]; the Lee cardiovascular complication risk score [19]; or the snoring, tiredness, observed apnea, blood pressure, BMI, age, neck circumference, and gender (STOP-BANG) obstructive sleep apnea screening score [20] in which 7 of the 8 items are collected from the patient’s responses to the conversational agent.

Access to the chatbot was made possible once the surgical decision was made, after which the patient received an email inviting them to create their personal account using a smartphone, tablet device, or computer. The data collected were editable at any time by the patient.

The MyRISK score was developed using the Delphi method [21]. The first step was to identify among all the items of data collected by the chatbot those that were relevant, that is, considered to have weight in the development of a predictive risk score. After 2 rounds of discussion, a panel of 6 experts reached a consensus on 16 items, which were then retained (Table 1). The second step involved defining the independent risk level (1, 2, or 3) of each of the 16 items (Table 1). The third step concerned developing an algorithm based on these 16 items to stratify the global perioperative risk level into 3 categories corresponding to a presumed low, intermediate, or high global perioperative risk. Briefly, patients were classified as low risk when all 16 criteria were level 1, as intermediate risk when ≥1 of the 16 criteria were level 2, and as high risk when ≥3 level 2 criteria or ≥1 level 3 criterion were present.

Finally, to make the MyRISK score a visual tool, a *green*, *orange*, or *red* dot was assigned to the low, intermediate, and high global perioperative risk levels, respectively. This color coding was easily accessible and visible on the digital dashboard of patients enrolled in the MAX program. Patients were then considered to have a *green*, *orange*, or *red* MyRISK score.

**Table 1:** MyRISK score criteria.

Criteria	Level 1	Level 2	Level 3
Age (years) [22]	<65	65 to 80	>80
BMI (kg/m^2^) [23]	<30	30 to 40	>40
Drug allergies	No	Yes	N/A^a^
Hemostasis disorders	No	Yes	N/A
Number of medications	0	1 to 5	>5
Active smoking [24]	No	Yes	N/A
Asthma	No	Yes	N/A
Sleep apnea syndrome [25]	No	Yes	N/A
Maximum level of activity (MET^b^) [26]	Walking up 2 flights of stairs without stopping; walking in the street (5-7 km/h); important domestic activities (washing the floor); and sports activities	N/A	Activities of daily living (meals and toileting); walking in the house; and walking in the street (3-5 km/h)
Cardiac symptoms during exercise	No	N/A	Yes
Hypertension	No	Yes	N/A
Cardiac disease [27]	No	Yes	N/A
Respiratory disease [24]	No	Yes	N/A
Renal disease [28]	No	Yes	N/A
Neurological disorders [29]	No	Yes	N/A
Diabetes	No	Yes	N/A

^a^N/A: not applicable.

^b^MET: metabolic equivalent.

### Postoperative Complications

Each patient was interviewed by telephone 6 months after surgery by one of the physicians involved in the study. After information was provided on study objectives and oral consent, the telephone survey was used to ask patients about the potential occurrence of postoperative complications. The survey was guided by a computerized structured questionnaire, allowing the secure collection of pseudonymized data. After oral consent, the computerized postoperative patient record form was consulted to ensure that there were no missing data concerning the occurrence of postoperative complications. The average duration of the interview was 9.8 (SD 9) minutes.

We considered that patients had a postoperative complication if they had experienced at least one adverse event among those listed in Textbox 1.

**List of adverse events considered.**

**Potential postoperative complications**

- Acute renal failure (creatinine increase ≥0.3 mg/dL [≥26.5 μmol/L] in 48 hours or creatinine increase ≥1.5×baseline creatinine in <7 days or diuresis <0.5 mL/kg/h for 6 hours or hospitalization for acute kidney injury) [30]
- Myocardial infarction (such as an increase in troponin associated with at least one of the following: signs of ischemia, ST-segment change, development of left branch block on electrocardiogram, or hospitalization for angina or myocardial infarction) [31-33]
- Acute heart failure (such as the presence of clinical, radiological, or echocardiographic signs; N-terminal pro brain natriuretic peptide elevation ≥900 pg/mL; or hospitalization for cardiac heart failure or cardiogenic pulmonary edema) [31,32]
- De novo atrial fibrillation (confirmed on electrocardiogram) [31,34]
- Transient ischemic attack or stroke (confirmed by computed tomography or magnetic resonance imaging or any hospitalization for stroke or transient ischemic attack) [35,36]
- Infection (such as fever requiring antibiotic therapy, hospitalization for fever, or suspected or documented infection)
- Respiratory complication (such as lung disease or respiratory compromise requiring oxygen or noninvasive or invasive ventilation or any hospitalization for respiratory compromise or lung disease) [37]
- Thromboembolic event (confirmed on Doppler ultrasound or computed tomography) [38]
- Hemorrhage (requiring transfusion)
- Rehospitalization
- Death

### User Satisfaction

Patient satisfaction with the quality and usability of the chatbot was assessed by the system usability scale (SUS). The SUS is a validated standardized tool for collecting users’ opinions on the perceived ease of use of a digitalized system [39,40].

Briefly, the SUS assesses user experience and acceptability (Table 2). Ten statements (5 positive and 5 negative) are listed. Users respond to each statement using a Likert scale ranging from 1 (*strongly disagree*) to 5 (*strongly agree*). The overall score is calculated to consider items with reversed valences. The final score is between 0 and 100; a score between 50 and 75 is considered fair, 75 to 85 is considered good, and >85 is considered excellent.

Patients’ overall satisfaction with the use of the digital conversational agent and with the course of the PAC (ie, face-to-face consultation or teleconsultation) was collected via a simple numeric rating scale (NRS) ranging from 0 (extremely dissatisfied) to 10 (extremely satisfied).

The anesthesiologists using the digital platform during this period were asked to assess their level of satisfaction by using the same evaluation scales (NRS and SUS).

**Table 2:** System Usability Scale (standard English version).

Statements	Scoring^a^				
	1	2	3	4	5
I think that I would like to use this system					
I found the system unnecessarily complex					
I thought the system was easy to use					
I think that I would need the support of a technical person to be able to use this system					
I found the various functions in the system were well integrated					
I thought there was too much inconsistency in this system					
I would imagine that most people would learn to use this system very quickly					
I found the system very cumbersome to use					
I felt very confident using the system					
I needed to learn a lot of things before I could get going with this system					

^a^Instruction on using the System Usability Scale: Please, circle the appropriate score for each statement from 1 (strongly disagree) to 5 (strongly agree).

### Machine Learning Model

Our goal was to train a machine learning model to predict the MyRISK score of patients having either a *green* or *red* score. This trained model was then asked to predict the MyRISK score of individuals classified as *orange* and reclassify them as either *modified green* or *modified red*.

#### Data Preprocessing

The data set used to train the model was extracted from a larger database of patients. We filtered out duplicates of individuals and features with >70% missing values. We also transformed nominal features following the *one-hot encoding* method, creating a new binary feature for each unique value. We finally filtered out individuals classified as *orange* for the prediction task. The final processed data set was composed of 1823 individuals classified as *green* or *red* for 83 features.

#### Feature Selection

To filter out redundant features, the recursive feature elimination (RFE) method was used [41]. Briefly, the following steps were applied: data were split into training and test sets; the model was trained on the training set, and its performance was evaluated on the test set; each feature contribution was evaluated, and the least contributing feature was identified (local Shapley additive explanations method [42]) and removed before going back to the first step.

**Figure 2:**
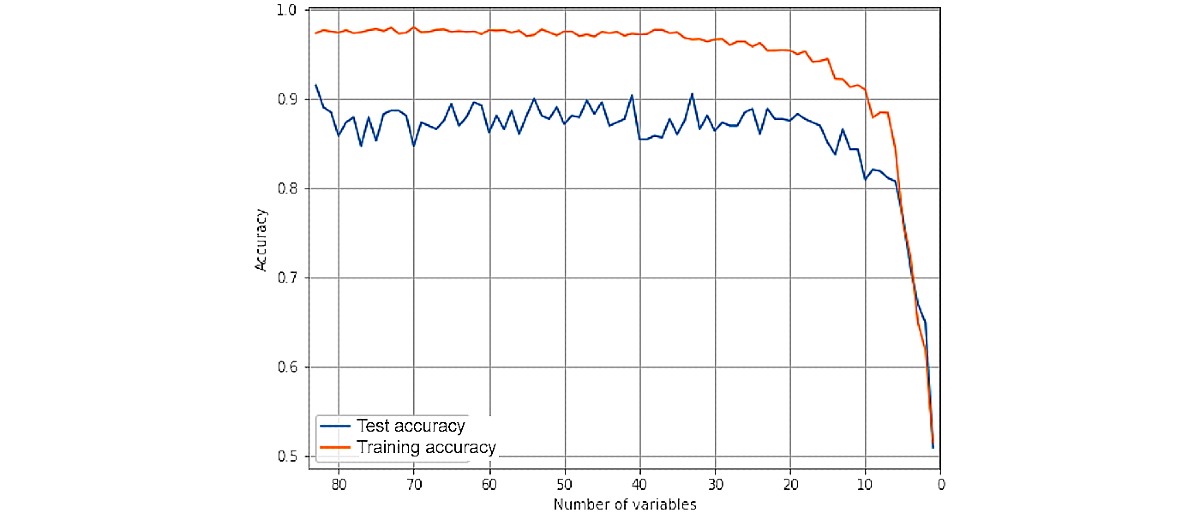
Evolution of the training and test accuracies of the model through the recursive feature elimination method. The test performance of the model starts to worsen significantly when <25 features are considered. The 25 most contributing features were finally selected for the final model training.

The RFE algorithm returns a list of model performances in the training and test sets for each feature. The evolution of the training and test accuracies of the model through the RFE are presented in Figure 2. In total, 25 features were finally selected in the data set for the final model training.

#### Model Training and Performance

The extreme gradient boosting classifying model was used to train the model on the 25 selected features [43]. Using a hyperparameter grid search, the training and test confusion matrices were obtained with good accuracy scores (97.5% and 96%, respectively).

All processing stages were performed using open-source software machine learning libraries sklearn 1.0.1 and extreme gradient boosting 1.5 (for the model) in Python programming language (version 3.9.7; Python Software Foundation).

### Statistical Analysis

Qualitative data were expressed as numbers (%). Quantitative data were expressed as median (IQR) or mean (SD) as appropriate. Categorical variables were compared using the Fisher exact test or the chi-square test. Quantitative variables were compared using the nonparametric Mann-Whitney *U* test. Multivariate analysis (stepwise logistic regression analysis) was performed to identify explanatory variables for the occurrence of postoperative complications at 6 months. The analysis was performed on an intention-to-treat basis. Statistical analysis was performed using MedCalc (version 12.6.1; MedCalc Software Ltd). *P*<.05 was considered statistically significant.

### Ethics Approval

This research is considered an experiment in educational sciences aiming to evaluate the participative and pedagogical quality of a new digital tool implemented in current practice. Hence, this research was deemed to fall outside the Jardé law, meaning that no formal ethics approval was required for this study.

Information about the participants’ health conditions was collected by the chatbot after they had created their account, but the company BOTdesign had access neither to the patients’ identity nor to their IP address. This strategy of data protection was decided in agreement with the eHealth committee of the University Hospital Center of Toulouse. The connection to the digital questionnaire was secure (following the *General Data Protection Regulation* guidelines). An email invitation to log in was sent to the patients after the appointment with the surgeon. Patients chose their own secret password to create their MAX account. Each patient was given oral and written information about this research before enrollment, ensuring that they did not have any objection to participating. This study did not present any risk for the participants and did not modify the usual care pathway or the time required for patient management.

## Results

### Population

Of the 1000 eligible patients who were scheduled for orthopedic surgery at the Toulouse University Hospital between June 1, 2020, and October 31, 2020, a total of 434 (43.4%) patients logged in to the chatbot. Of these 434 patients, 401 (92.4%) agreed to participate in this study. The characteristics of the studied population are presented in Table 3.

**Table 3:** Demographic characteristics of the patients.

Characteristics		Values
Age (years), median (IQR)		39 (27-54)
**Sex (n=401), n (%)**		
	Male	241 (60.1)
	Female	160 (39.9)
BMI (kg/m^2^), mean (SD)		25 (4)
**Education (n=388), n (%)**		
	Undergraduate	83 (21.4)
	Graduate	135 (34.8)
	Postgraduate	170 (43.8)
Ambulatory care pathway (n=388), n (%)		297 (76.5)
**Surgical risk (n=389), n (%)**		
	Minor	284 (73)
	Intermediate	89 (22.9)
	Major	16 (4.1)
**ASA^a^ score (n=389), n (%) **		
	1	282 (72.5)
	2	89 (22.9)
	3	18 (4.6)
	4	0 (0)
**MyRISK score (n=389), n (%)**		
	Green (low risk)	100 (25.7)
	Orange (intermediate risk)	150 (38.6)
	Red (high risk)	139 (35.7)
STOP-BANG^b^ score modified (out of 7), median (IQR)		1 (1-2)
Lee score modified (out of 4), median (IQR)		0 (0-1)
APAIS^c^ anesthesia score (out of 15), median (IQR)		5 (3-7)

^a^ASA: American Society of Anesthesiologists.

^b^STOP-BANG: snoring, tiredness, observed apnea, blood pressure, BMI, age, neck circumference, and gender.

^c^APAIS: Amsterdam Preoperative Anxiety and Information Scale.

### User Satisfaction

The median satisfaction score of patients regarding the use of the chatbot as assessed by the NRS was 8.0 (IQR 7.0-9.0) out of 10. The median satisfaction score regarding the use of the digital questionnaire as assessed by the SUS was 90.0 (IQR 82.5-95.0) out of 100.

The median SUS score was higher for users who chose a tablet device or smartphone (97/391, 24.8%) than for those who chose a computer (294/391, 75.2%): 92.5 (IQR 85.0-97.5) versus 90.0 (IQR 82.0-95.0), respectively (*P*=.01).

A large majority of the PACs were teleconsultations (331/401, 82.5%). Regarding the patients’ wishes for a future PAC if indicated, 54.7% (181/331) of the patients who received a teleconsultation wished to keep this mode of PAC in the future, whereas 19% (13/70) of the patients who received a face-to-face consultation wished to keep the same mode of PAC (*P*=.08). The mean patient satisfaction score (NRS) regarding the teleconsultation was 8.4 (SD 1.59) out of 10.

The satisfaction score of the anesthesiologists (n=18) regarding the use of the digital platform was collected. Their median satisfaction score was 7.0 (IQR 6.0-8.0) out of 10, and their median SUS usability score was 72.5 (IQR 63.1-88.1) out of 100.

### Postoperative Complications

Of the 389 patients analyzed, 16 (4.1%) had a postoperative complication at 6 months. No deaths were reported. The results of the univariate analysis are presented in Table 4.

A dependency relationship between the ASA and MyRISK scores was found (Table 5).

Compared with ASA score=1, an ASA score of ≥3 was independently associated with the occurrence of postoperative complications at 6 months (odds ratio [OR] 5.8, 95% CI 1.7-20.2; *P*=.006). In comparison with a *green* score, a *red* score was independently associated with the occurrence of postoperative complications at 6 months (OR 5.9, 95% CI 1.5-22.3; *P*=.009). Age and surgical risk included in the analysis were not identified as independent variables of the occurrence of postoperative complications. The area under the receiver operating characteristic curve (AUC) of the selected model was 0.78 (95% CI 0.73-0.82).

Finally, a *red* score predicted postoperative complications with 75% sensitivity, 98% negative predictive value, 66% specificity, and 9% positive predictive value (AUC=0.70).

**Table 4:** Comparison between patients with postoperative complications and those without postoperative complications (univariate analysis; N=389).

			No postoperative complications (n=373)		Postoperative complications (n=16)		*P* value	
Age (years), median (IQR)			39.0 (27.0-53.0)		56.5 (44.0-68.0)		.007	
Ambulatory care pathway, n (%)			292 (78.3)		5 (31.2)		<.001	
**Surgical risk, n (%)**								.06
	Minor	276 (74)		8 (50)				
	Intermediate	83 (22.2)		6 (37.5)				
	Major	14 (3.8)		2 (12.5)				
**ASA^a^ score, n (%)**								<.001
	1	277 (74.3)		5 (31.2)				
	2	83 (22.2)		6 (37.5)				
	3	13 (3.5)		5 (31.2)				
	4	0 (0)		0 (0)				
**MyRISK score, n (%)**								.002
	Green (low risk)	100 (26.8)		0 (0)				
	Orange (intermediate risk)	146 (39.1)		4 (25)				
	Red (high risk)	127 (34.1)		12 (75)				
Number of medications, median (IQR)			0.0 (0.0-2.0)		0.5 (0.0-1.0)		.65	
Active smoking, n (%)			172 (46)		8 (50)		.80	
Asthma, n (%)			43 (11.5)		2 (12.5)		.99	
Sleep apnea syndrome, n (%)			14 (3.7)		2 (12.5)		.14	
Cardiovascular disease, n (%)			24 (6.4)		3 (18.7)		.09	
Renal disease, n (%)			7 (1.8)		0 (0)		.99	
Neurological disease, n (%)			26 (6.9)		2 (12.5)		.33	
Digestive disease, n (%)			55 (14.7)		3 (18.7)		.72	
Diabetes, n (%)			19 (5)		1 (6.2)		.58	
APAIS^b^ anesthesia score (out of 15), median (IQR)			5.0 (3.0-7.0)		6.0 (4.0-7.5)		.61	
Lee score modified (out of 4), median (IQR)			0.0 (0.0-0.0)		0.0 (0.0-0.0)		.52	
Apfel score modified (out of 3), median (IQR)			1.0 (0.0-1.0)		1.0 (0.0-2.0)		.48	
STOP-BANG^c^ score modified (out of 7), median (IQR)			1.0 (1.0-2.0)		2.0 (1.0-2.5)		.46	

^a^ASA: American Society of Anesthesiologists.

^b^APAIS: Amsterdam Preoperative Anxiety and Information Scale.

^c^STOP-BANG: snoring, tiredness, observed apnea, blood pressure, BMI, age, neck circumference, and gender.

**Table 5:** Correlation between American Society of Anesthesiologists (ASA) and MyRISK scores (N=400).

MyRISK score	ASA score, n (%)				*P* value
	1	2	3		
Green (low risk)	98 (24.4)	7 (1.7)	0 (0)	<.001	
Orange (intermediate risk)	124 (30.9)	28 (7)	1 (0.2)	<.001	
Red (high risk)	66 (16.5)	58 (14.5)	18 (4.5)	<.001	

### Recalculation of the Predictive Value of the MyRISK Score Using a Machine Learning Model

#### Reclassification of Patients With an Orange MyRISK Score

Among the 389 patients analyzed for the primary end point, 150 (38.6%) were initially classified as *orange*. Of these 150 patients, 4 (2.7%) experienced postoperative complications. Of the 146 patients classified as *orange* with no postoperative complications, 65 (44.5%) were reclassified as *modified red* and 81 (55.5%) as *modified green* using the trained model. Similarly, of the 4 patients with postoperative complications, 3 (75%) were finally reclassified as *modified red* and 1 (25%) as *modified green*.

**Figure 3:**
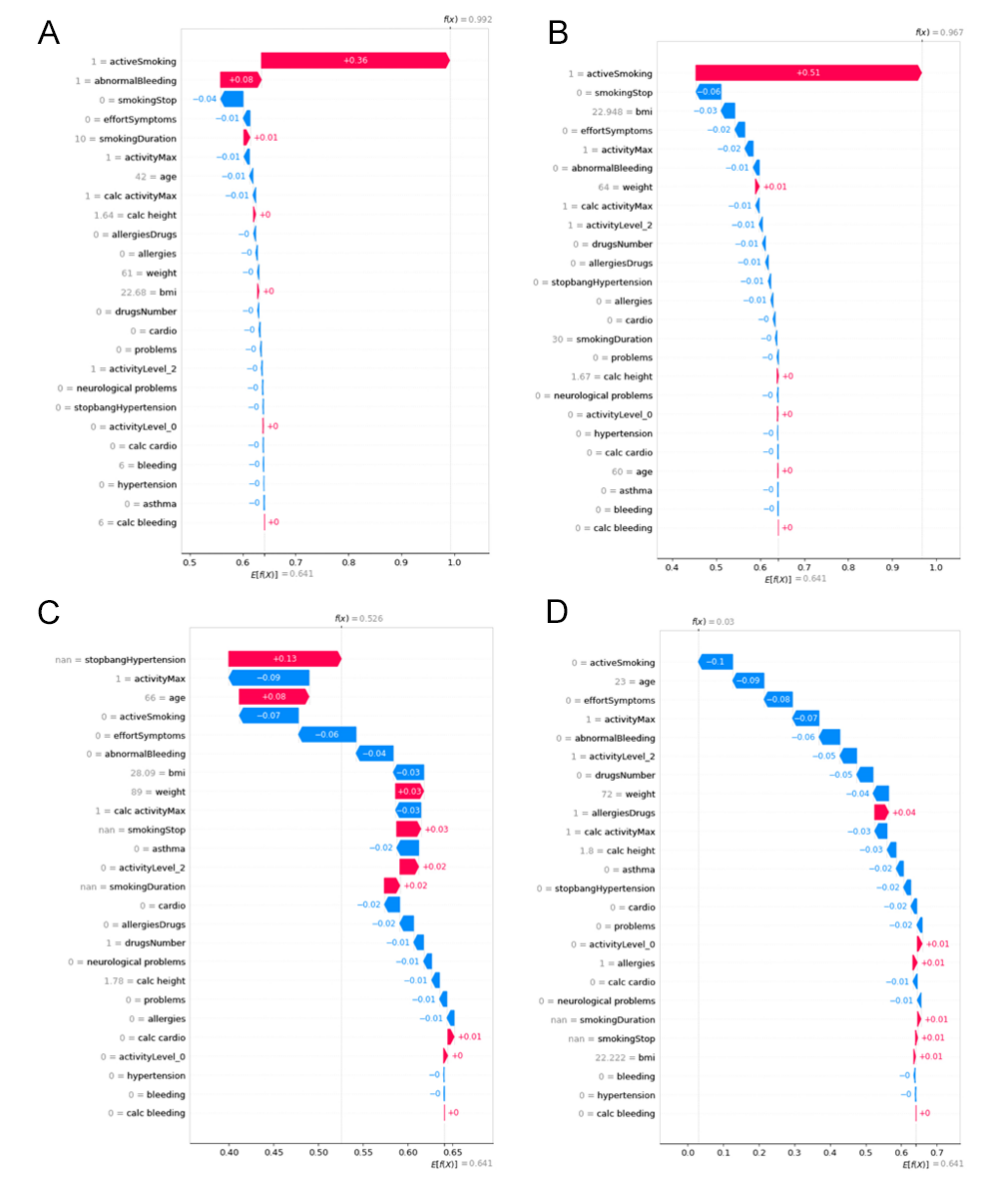
Computation of the contribution of each of the 25 features (local Shapley additive explanations method) of the 4 patients with an orange MyRISK score who experienced postoperative complications. The first 3 patients (represented by A, B, and C) were reclassified as modified red by the machine learning model; (D) represents the patient who was finally predicted modified green.

Concerning these 4 patients, the contribution of each feature was computed with the local Shapley additive explanations method (refer to the Methods section for details). The results are presented in Figure 3.

#### Predictive Value of the Modified MyRISK Score

Once the 4 patients classified as *orange* were reclassified, a *modified red* MyRISK score was identified as strongly associated with postoperative complications at 6 months (OR 21.8, 95% CI 2.8-171.5; *P*=.003). An ASA score of ≥3 was also associated with postoperative complications (OR 4.7, 95% CI 1.4-16; *P*=.01).

Finally, a *modified red* score predicted postoperative complications with high sensitivity (94%) and high negative predictive value (99%) but with low specificity (49%) and very low positive predictive value (7%; AUC=0.71).

## Discussion

### Principal Findings

Through this study, we were able to validate the prognostic predictive value of a perioperative risk score established from data collected by a digital conversational agent implemented to assist the anesthetist during the PAC. In this context, the MyRISK score was correlated with the incidence of complications occurring in the first 6 months postoperatively. The strength of its predictive value was increased using a machine learning model to reclassify patients classified as intermediate risk. The use of an objective method allowing perioperative risk stratification according to a color code (ie, visual tool) and available before the PAC could be relevant for physicians. Finally, the use of this innovative digital tool seems to fully satisfy users.

We were able to identify a dependency between the level of perioperative risk at the end of the medical clinical evaluation (ie, ASA score) and the one calculated digitally before the PAC (ie, MyRISK score). This correlation had already been found by Zuidema et al [44] in a 2011 study using a 22-item numerical questionnaire administered to 14,349 patients, the authors highlighted that a computerized risk assessment could perform well and correlate with the clinical assessment of the ASA score (AUC=0.953), while limiting the interindividual variability of a clinician-assessed ASA score. In this context, it is interesting to note that the primary factor in numerical misclassification of the ASA score was an incomplete or incorrect patient response. More recently, Enneking et al [45] presented a 5-criteria composite preoperative risk score (*patient-centered anesthesia triage system score*) that correlated well with the ASA score (AUC=0.75, 95% CI 0.69-0.83), highlighting its usefulness for patient triage. Since 2017, the authors have been using this score in clinical practice to propose the systematic performance of a teleconsultation for patients classified as no risk.

By analyzing complications occurring in the first 6 postoperative months, we were able to validate the independent prognostic predictive value of the MyRISK score on the occurrence of serious postoperative adverse events. To our knowledge, this approach of validating a numerical risk score on objective criteria (ie, postoperative complications) has never been described in the literature. Thus, by reliably classifying patients according to their level of perioperative risk, the use of the (modified) MyRISK score could allow, before the PAC, triaging of patients by proposing the most appropriate modality of consultation (eg, teleconsultation for patients classified as [modified] green and face-to-face consultation for patients classified as [modified] red). Secondary benefits linked to this triage modality are expected: patients’ experience could be improved by reducing waiting time and optimizing consultation time. In addition, the face-to-face consultation could be dedicated to the management of patients with the most complex conditions who require, for example, specialized examinations or consultations. Moreover, as the role of anesthesiologists in the postoperative management of patients is growing, the implementation of a postanesthetic consultation could be a future trend, particularly for patients classified as high risk. In this setting, digital tools help to keep patients and caregivers connected for better follow-up, allowing an early detection or even prevention of postoperative complications.

The current health context has temporarily established the need to reduce travel throughout the country to limit interpersonal contact. Thus, the French National Authority for Health has recommended the use of telemedicine to enable remote management of patients [46]. Finally, the COVID-19 pandemic has highlighted the potential of digital health solutions in facilitating the adaptation of the organization of care in a crisis situation [47]. In this context, the development of the digital solution *MAX* helped us to organize the resumption of surgical activity when restrictions were lifted. A relevant distribution of patients between teleconsultation and face-to-face consultation using the (modified) MyRISK score could allow the indefinite continuance of teleconsultation after the pandemic. However, only 25.7% (100/389) of the patients were classified as low perioperative risk (ie, *green*). Questions still remain as to the proper organizational management of patients classified as intermediate risk (ie, *orange*). It is worth noting that the percentage of patients classified as low perioperative risk (theoretically eligible for teleconsultation) increases to 47% (183/389) when using the modified classification.

In our study, patient satisfaction with the use of the digital questionnaire as well as the PAC process was excellent. The developed digital conversational agent seems to be an adequate platform for the collection of patients’ medical information, as shown by the excellent usability score obtained. Moreover, the usability seems to be better when completing the digital questionnaire on a smartphone or tablet device. Our results highlight the enthusiasm of patients for using a digital health platform. These results are in agreement with those described by VanDenKerkhof et al [14], where patient comfort was increased by >70% by computerizing the preanesthetic questionnaire.

Although we did not strictly evaluate the reliability of the data collected by the digital conversational agent, Osman et al [48] demonstrated a response reliability of >90% when a computerized preanesthetic questionnaire was used. Thus, there is consensus in the literature now of the reliability of digital collection of information [49].

Several factors may have favored patient acceptance of this new digital solution. The young age of the patients enrolled (median age 39.0, IQR 27.0-54.0 years) and their level of education (305/389, 78.4%, had graduate or postgraduate degrees) probably explain the very high levels of satisfaction and usability obtained. These results are in agreement with those obtained by Kruse et al [50]. Indeed, the age, level of education, and computer skills of the patients were the 3 main barriers to telehealth adoption identified by the authors [50]. The acceptability and satisfaction of the patients obtained during the use of this digital support encourages our department to develop telemedicine solutions. This enthusiasm is reinforced by the good satisfaction ratings provided by the members of the medical team during the use of this platform, which is probably linked to the automatic integration of the data collected by the digital conversational agent into the computerized PAC file. However, a qualitative analysis of the main difficulties encountered by the physicians highlighted the absolute necessity of good interoperability among the various software systems.

In our study, the rate of postoperative complications observed at 6 months was 4.1% (16/389). No deaths were recorded. These results are in accordance with those already published in the literature. Indeed, in 2013, Chikuda et al [51] evaluated postoperative morbidity and mortality rates in scheduled orthopedic surgery among >100,000 patients [51]. In this context, the morbidity and mortality rates were 4.2% and 0.11%, respectively.

Our study includes several limitations. First, the results obtained in preoperative scheduled surgeries cannot be extrapolated to the context of urgent surgeries where the use of a digital conversational agent to assist the PAC seems difficult to achieve. Second, 82.5% (331/401) of the PACs analyzed in the study were teleconsultations. The period when the patients were included corresponded to the end of the first lockdown, which explains this high rate of teleconsultations that is not very representative of the subsequent evolution of the practices of our unit (approximately 40% currently). Third, the potential benefits of allocating patients to teleconsultation or face-to-face consultation according to the MyRISK score deserve to be studied in more detail; for example, analysis of patient-perceived quality-of-care indicators (eg, patient-reported outcome measures and patient-reported experience measures) [52] could demonstrate that the experience of care perceived by patients classified as *green* receiving teleconsultation is optimal, notably by avoiding unnecessary travel that disrupts their personal and professional schedules. Fourth, collection of the overall consumption of care by consulting the national health data system could have increased the prognostic predictive value of the MyRISK score by a more global and exhaustive analysis of the postoperative evolution of patients. This type of medicoeconomic approach should be favored in the future. Fifth, the experimental design of our study did not allow us to evaluate the reasons for the nonconnection to MAX by a significant number of patients. Thus, it seems likely that the satisfaction and usability scores were overestimated because they were collected only from the user population of patients. Analysis of the overall data is fundamental to understanding the potential explanatory factors of the digital divide in this context. Fifth, further studies are needed to extend the validation of the MyRISK score to other surgical populations and thus generalize our results. Finally, there is a need for studies examining the impact that the use of these tools has on clinical decision-making and patient outcomes.

### Conclusions

To conclude, we were able to demonstrate the prognostic value of a perioperative risk score established from data collected by a digital conversational agent implemented before the PAC. In this setting, the MyRISK score was associated with the occurrence of postoperative complications at 6 months after surgery. The strength of its predictive value was increased using a machine learning model to reclassify patients classified as intermediate risk. The excellent levels of satisfaction and usability obtained from patients encourage us to develop and use this digital solution in health care. Further studies evaluating the overall use of the MyRISK score are necessary before using this digital stratification method to guide patients to teleconsultation or face-to-face consultation or to provide a perioperative personalized care pathway for patients at highest risk.

## Appendix

Multimedia Appendix 1 Patient responding to the preanesthetic digital conversational agent.

Multimedia Appendix 2 Example of patient completing the digital questionnaire.

## Abbreviations

ASA: American Society of Anesthesiologists
AUC: area under the receiver operating characteristic curve
MAX: Medical Assistant Experience
NRS: numeric rating scale
OR: odds ratio
PAC: preanesthetic consultation
RFE: recursive feature elimination
STOP-BANG: snoring, tiredness, observed apnea, blood pressure, BMI, age, neck circumference, and gender
SUS: system usability scale
