# Supplementary figures and images for: Perioperative Risk Assessment of Patients Using the MyRISK Digital Score Completed Before the Preanesthetic Consultation: Prospective Observational Study
Source: JMIR Perioper Med. 2023 Jan 16;6:e39044. doi: 10.2196/39044 (PMC9887512; doi:10.2196/39044)

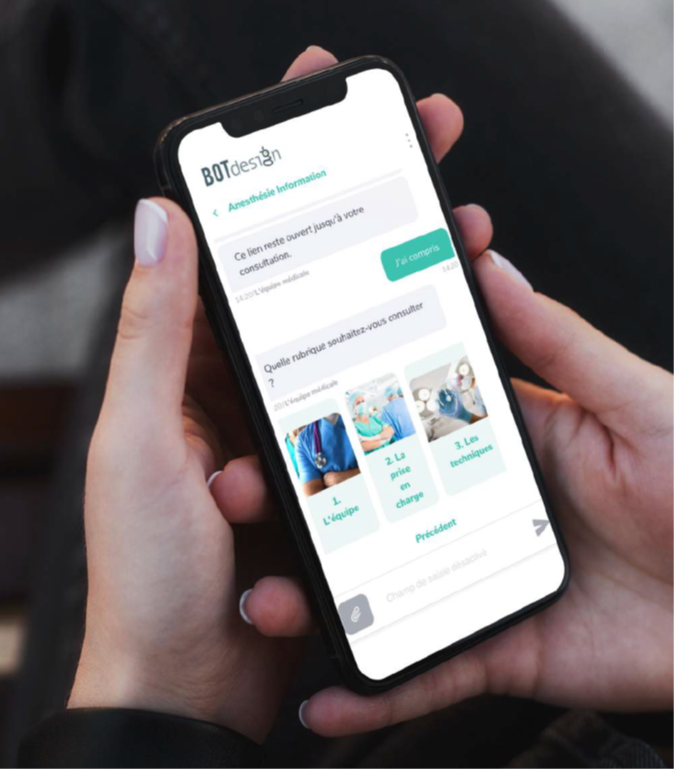

Supplement: Multimedia Appendix 1 [file periop_v6i1e39044_app1.png]
